# Parotid fistula secondary to suppurative parotitis in a 13-year-old girl: a case report

**DOI:** 10.1186/1752-1947-4-249

**Published:** 2010-08-05

**Authors:** Amith I Naragund, Vijayanand B Halli, Ramesh S Mudhol, Smita S Sonoli

**Affiliations:** 1 Department of ENT and HNS, Jawaharlal Nehru Medical College, KLE University, Belgaum, India; 2 Department of Biochemistry, Jawaharlal Nehru Medical College, KLE University, Belgaum, India

## Abstract

**Introduction:**

The most common cause of parotid fistula is trauma, followed by malignancy, operative complications (parotidectomy or rhytidectomy) and infection. Acute suppurative parotitis can rarely produce parotid fistula. There are various treatment options available, however it is necessary to standardize the treatment according to the duration of history and the patient's general condition.

**Case report:**

A 13-year-old Indo-Caucasian girl presented to us with a two-year history of clear watery discharge from a wound just above and behind the angle of her right jaw. A diagnosis of salivary (parotid) fistula was made based on clinical examination and investigations. The parotid fistula was successfully managed.

**Conclusion:**

Parotid fistula secondary to suppurative parotitis is rare and difficult to manage successfully. Meticulous dissection, complete excision of the fistulous tract with closure of the parotid fascia and layered closure of the incision followed by application of a post-operative pressure bandage, anticholinergic agents and antibiotics contribute significantly to the successful management of this difficult clinical condition.

## Introduction

A parotid fistula is a communication between the skin and a parotid duct or gland through which saliva is discharged [1]. The most common cause of parotid fistula is trauma, followed by malignancy, operative complication (parotidectomy or rhytidectomy) and infection [2,3]. Acute suppurative parotitis can rarely produce a parotid fistula. Flow through the fistula increases during meals, particularly during mastication, which confirms diagnosis [1]. A rare case of parotid gland fistula following suppurative parotitis is described here.

## Case report

A 13-year-old Indo-Caucasian girl came to our hospital with a history of clear watery discharge from a wound just above and behind the angle of her right jaw for two years. The discharge increased while eating food and chewing. Her medical history revealed a swelling just behind her right jaw associated with a throbbing type of pain and fever two years ago, which burst open with pus discharge. A week later, she started getting a clear watery discharge from the affected site.

**Figure 1:**
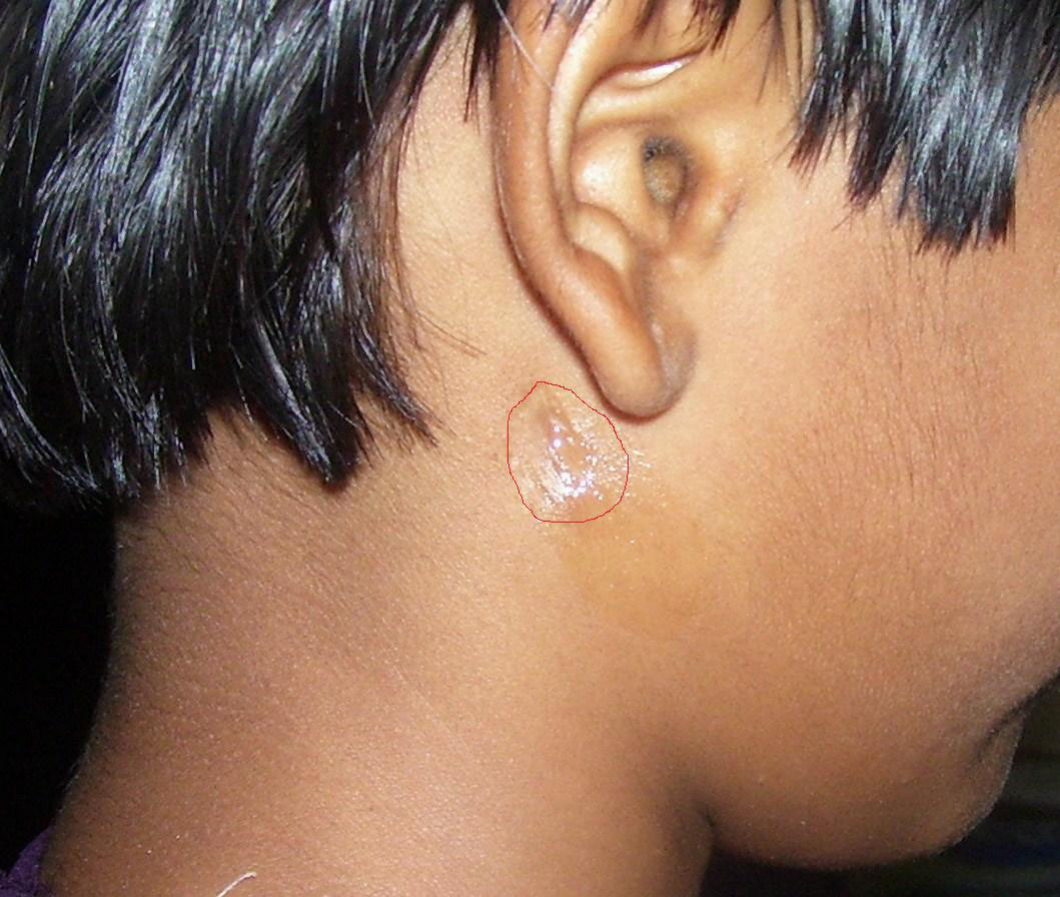
**Pre-operative picture of parotid fistula with leakage of serous fluid from the fistulous tract and scarring of surrounding area (red circle)**.

On examination, there was a pinpoint size opening just posterosuperior to the angle of the mandible with a continuous dribbling of clear serous fluid and scarring of the surrounding area (Figure 1). Laboratory analysis of the fluid revealed raised salivary amylase levels (7800 IU/mL), which confirmed the diagnosis of a salivary fistula. Our patient was successfully managed by a simple surgical technique, described below.

**Figure 2:**
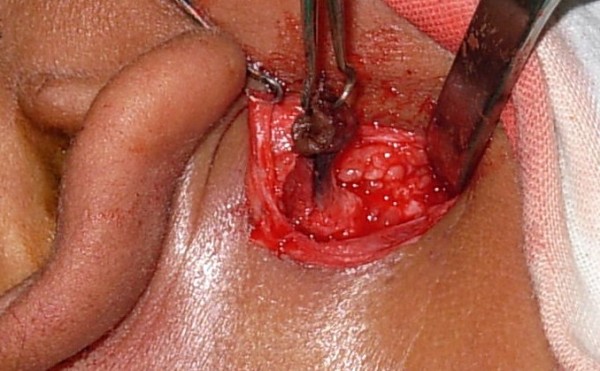
**Intra-operative picture of fistulous tract containing methylene blue dye**.

**Figure 3:**
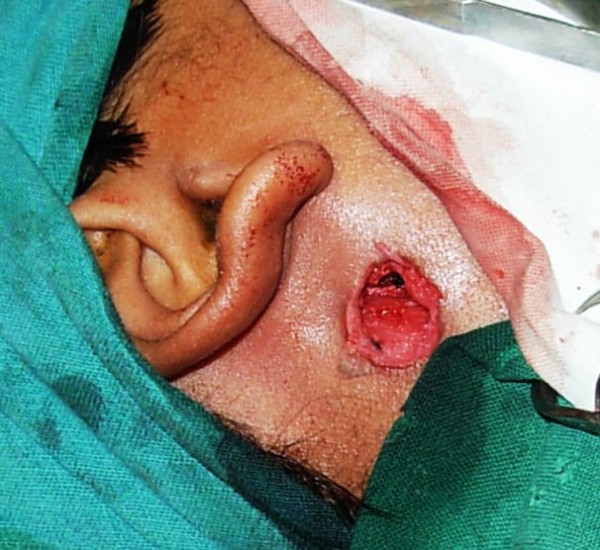
**Fistulous tract completely excised by opening superficial parotid fascia**.

**Figure 4:**
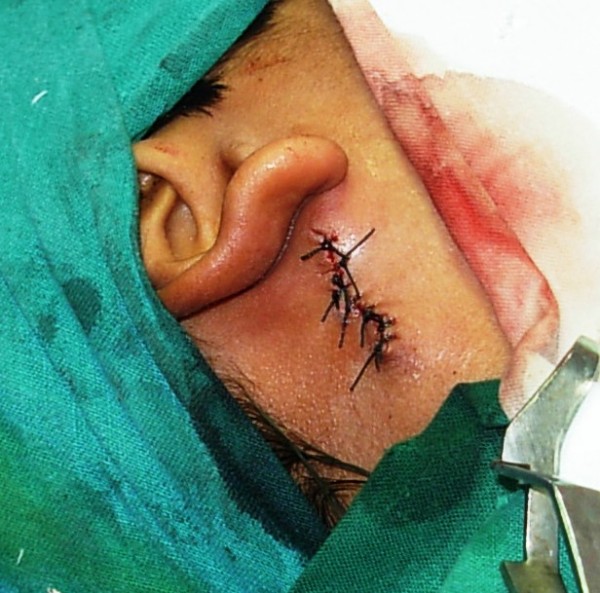
**Skin incision closed with 3-0 silk sutures**.

The procedure was performed under general anesthesia with local infiltration of 1 in 100,000 adrenaline around the fistulous opening to minimize intra-operative bleeding. Methylene blue was then injected into the fistulous opening using a 26-gauge needle (blunt tip) under microscopic magnification. The dye was seen exiting from the natural opening of the Stenson's duct, indicating a patent ductal system. An elliptical incision of 1 cm diameter was taken around the fistulous opening, which included the scar tissue. The skin island was then held with skin hooks and the subcutaneous tissue dissected until the fistulous tract containing dye was visible (Figure 2). The fistulous tract was then traced proximally until it entered the thick parotid fascia. The fascia was then incised and the tract was seen entering the superficial lobe of parotid. It did not extend up to branches of the facial nerve. At this level, the superficial lobe of parotid was carefully dissected and the fistulous tract was completely excised (Figure 3). The parotid fascia was approximated and sutured with 3-0 vicryl and the wound closed in layers. The skin was closed using 3-0 silk sutures (Figure 4) and a tight pressure dressing applied. Following surgery, there was no facial nerve deficit.

**Figure 5:**
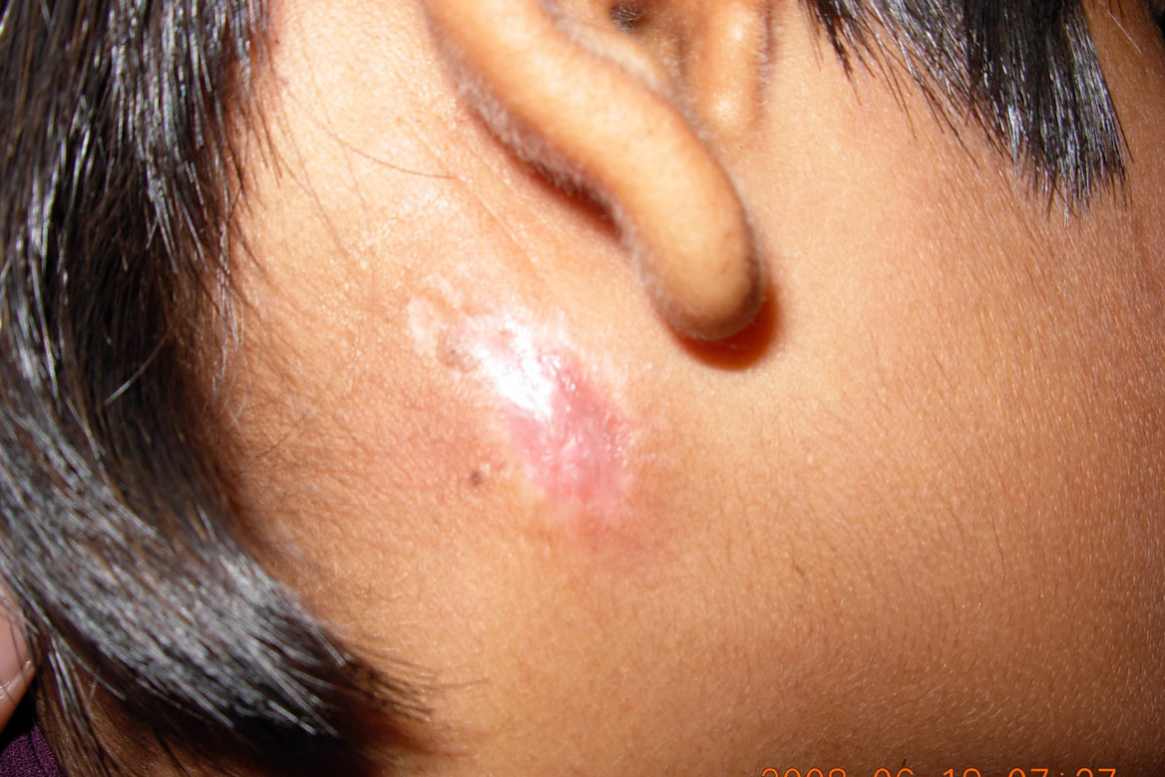
**Post-operative picture after 3 months showing successful closure of fistulous tract**.

Post-operatively, our patient was kept on nil by mouth for 24 hours and put on intravenous fluids, antibiotics, atropine and analgesics. Our patient was discharged on oral antibiotics and analgesics on the third post-operative day. Her sutures were removed on the seventh day. Histopathological examination of the fistulous tract showed no underlying malignancy or evidence of any specific (granulomatous) disease. Our patient was followed up three months later and was found to have successful healing of her wound with no complications or recurrence (Figure 5).

## Discussion

Parotid fistula can rarely occur as a complication of acute suppurative parotitis, as in this case. The diagnosis is made by combining information from the patient's history with findings from clinical examination, which in our case revealed a small opening over the skin with discharge of clear serous fluid that increases during ingestion of food and mastication. In doubtful cases fluid can be sent for laboratory analysis; raised salivary amylase levels confirm the diagnosis [1]. Computed tomography fistulography can be performed to look for the extent of the fistula [4]. Several operative and conservative treatments have been described for parotid gland fistulae, but to date no method is satisfactory [5,6]. Early fistulae are self-limiting and usually respond to conservative management by reducing the salivary secretions (anti-cholinergics) and application of a pressure dressing. In cases of failure of conservative management or in delayed presentations, management is either injection of botulinum A toxin into the gland or surgery. The surgical option includes either tympanic neurectomy, or fistulectomy with or without superficial parotidectomy [2,3]. The major secretomotor fibers to the salivary gland are cholinergic parasympathetic and are susceptible to inhibition by the botulinum toxin. The localized cholinergic block achieved with botulinum toxin injections avoids the side effects caused by systemic anti-cholinergic drugs and avoids surgical risks [5]. Inhibition of parotid secretion leads to a temporary block in salivary flow, followed by glandular atrophy, thus allowing healing of the fistula [1]. Another form of treatment is tympanic nerve section, which has a low success rate and can take a long time to achieve healing of the fistula [1]. The results of the latter two techniques are comparatively slow and unpredictable [6].

In the case of our patient, as it was a delayed presentation, a fistulectomy was performed. The superficial lobe of parotid was dissected carefully to prevent trauma, which could cause further salivary leak leading to the formation of sialocele and a recurrent fistula [5]. The wound was closed tightly and a pressure dressing applied. Histopathological examination of the fistulous tract was performed, as rarely there can be underlying malignancies or chronic granulomatous lesions associated with the condition. Surgical excision of the fistulous tract followed by tight pressure dressing of the wound is an effective management option, as in our patient.

## Conclusions

Parotid fistula occurring as a complication of acute suppurative parotitis is rare and difficult to manage successfully. Meticulous dissection, complete excision of the fistulous tract with closure of the parotid fascia and layered closure of the incision, followed by post-operative pressure bandage application, anti-cholinergic agents and antibiotics contributed significantly to the successful management of this difficult clinical condition.

## Consent

Written informed consent was obtained from the patient's guardian for publication of this case report and any accompanying images. A copy of the written consent is available for review by the Editor-in-Chief of this journal.

## Competing interests

The authors declare that they have no competing interests.

## Authors' contributions

AIN drafted the article, performed the literature search, compiled the data, and acquired the images cited in this case report. VBH and RSM reviewed and edited the manuscript. SSS supervised the manuscript and helped in biochemical analysis. All authors read and approved the final manuscript.
